# Time-dependent expression of high-mobility group box-1 and toll-like receptors proteins as potential determinants of skin wound age in rats: Forensic implication

**DOI:** 10.1007/s00414-022-02788-z

**Published:** 2022-02-07

**Authors:** Yasmina M. Abd-Elhakim, Bothina H. F. Omran, Shimaa A. Ezzeldein, Amany I. Ahmed, Nabela I. El-Sharkawy, Amany Abdel-Rahman Mohamed

**Affiliations:** 1 Department of Forensic Medicine and Toxicology, Faculty of Veterinary Medicine, Zagazig University, Zagazig, Egypt; 2 Department of Forensic Medicine and Clinical Toxicology, Faculty of Human Medicine, Zagazig University, Zagazig, Egypt; 3 Department of Surgery, Anesthesiology and Radiology, Faculty of Veterinary Medicine, Zagazig University, Zagazig, Egypt; 4 Department of Biochemistry, Faculty of Veterinary Medicine, Zagazig University, Zagazig, Egypt

**Keywords:** High-mobility group box-1, Toll -like receptors, Pro-inflammatory cytokine, Nitric oxide, Wound age estimation, Real-time PCR

## Abstract

The skin wound age determination in living subjects is an imperative task for forensic experts. In this study, we investigated the time-dependent expression of high-mobility group box-1 (HMGB1) and toll-like receptors 2 and 4 (TLR2 and 4) in rat skin wounds using real-time PCR and seek their forensic potentials during the skin wound repair process. In addition, the levels of serum pro-inflammatory cytokines (tumor necrosis factor-alpha (TNF-α) and interleukin 6 (IL-6)), as well as nitric oxide (NO) production, were measured. The wound tissue and serum samples were collected after 30 min, 2 h, 6 h, 12 h, 1 day, 3 days, 5 days, and 7 days after incision. As a control (zero time), skin specimens and blood samples were collected without incision. The results reveal that the HMGB1, TLR2, and TLR4 expression levels were increased in a time-dependent manner until the first day where the peak level was achieved for the three tested genes compared with the zero time. On the 7^th^ day, the statistical significance was lost for TLR2 and TLR4 but persisted for HMGB1. The serum TNF-α, IL6, and NO levels peaked within 30 min and 1^st^ and 3^rd^ day after injury, respectively. On the 7^th^ day after incision, no significant differences exist in the TNF-α serum level compared to the control group, but the statistical significance persisted for IL6 and NO. It was apparent that the analyzed genes in the wound tissues showed higher R2 values rather than the serum biochemical indicators. Of note, a strong positive correlation was evident between the HMGB1 and that of TLR2 and TLR4 relative expression as well as IL-6 serum level. Conclusively, based on the observed changes in the analyzed markers in wound tissues and serum and R2 values obtained from mathematical models established to determine the wound age, the relative expression of HMGB1, TLR2, and TLR4 could be a reliable indicator for wound age determination in living subjects. Further investigation of these markers and mathematical models in human tissues is necessary.

## Introduction

In forensic medicine, the determination of the skin wound’s age in living subjects is an extremely important issue [1]. When physical abuse victims report a crime, a forensic medical officer shall be consulted to assess the injury, including the estimate for the wound age. But, the macroscopic description of a wound is known to be inadequate in determining the age of the wound [2, 3]. In forensic autopsies, numerous immunohistochemical markers, in addition to traditional histological features of wound healing, are used to define wound age more precisely [4–7]. To that end, skin excisions are performed in order to assess the actual damage which occurs primarily at the dermo-subcutaneous junction [8]. Nevertheless, it is unethical to excise skin wounds in living subjects[1]. Thus, more appropriate methods and potential determinants to define the age of injury in living subjects are therefore required.

The wound healing process, including skin wounds, has been characterized into three phases including inflammation, proliferation, and maturation [2]. Neutrophilic granulocytes are the first inflammatory cells to invade the site of wound. They can be extravascular detected after 20–30 min of infliction in human skin wounds [6, 9]. Lymphocytes attracted by chemokine to the wound can be found as early as 5–6 h after injury [10]. Macrophages can also be found 5–6 h after injury, although they were reported to peak in wounds 1–2 days as recorded in animal models and human skin [11]. During this course, several mediators are released from these cells.

High mobility group box 1 (HMGB1), known as amphoterin, is mainly a nuclear protein found in various eukaryotic cells and has an amino-acid sequence among species that has been highly conserved. HMGB1 has two separate functions in cellular systems. First, it acts as an intracellular transcription regulator with a critical role in maintaining the function of DNA [12]. Second, HMGB1 is translocated in all eukaryotic cells to outside of the nucleus after necrosis and released from the macrophage via activation of tumor necrosis factor alpha (TNF-α), lipopolysaccharides (LPs), interferon (IFN)-γ, and interleukin (IL)-1 [13–15]. It was recently recognized as a “late” inflammatory mediator [16]. It is extracellular secreted by macrophages and activated monocytes or passively released by necrotic or injured cells [17]. HMGB1 interaction with the receptor for advanced glycation end products (RAGE) and the toll-like receptor- 2 (TLR2), and TLR4 has been reported. The former interaction subsequently induced extracellular signal-regulated signaling, which triggers cytokine production [18, 19]. TLRs are a class of immunological pattern recognition receptors [20].

Substantial evidence now demonstrate that many different cell types, including inflammatory infiltrating or residents, fibroblasts, keratinocytes, and EC, are expressing specific TLRs types [21]. In particular TLR2 and 4 are expressed in several types of cells including neutrophils [22], macrophages [23], CD4^+^, CD8^+^[24], fibroblasts [25], and adipocytes [26]. Chakraborty et al. [27] reported that HMGB1 unregulated the TLR2 and TLR4 surface receptors, which in turn increase nitric oxide (NO) production which is a short-lived radical previously shown to be synthesized in wounds [28]. In spite of the certain TLRs role in controlling the immune reaction in acute wound healing, the TLR functions in chronic wound healing are poorly understood [29].

In the forensic context, Kikuchi et al. [30] confirmed possible use of serum HMGB1 for the postmortem interval estimation. However, up till know, there are no available report on the probable use of association between HMGB1 and TLR2 and 4 as a determinant of chronological estimation of wounds in living subjects. Thus, this study aimed to assess the effectiveness of combined assessment of wound age with the three markers HMGB1, TLR2, and TLR4 in a time-course experiment in a rat model using molecular means. Moreover, the correlation of the earlier markers with the serum pro-inflammatory cytokines levels (TNF-α and IL-6) and NO production has been assessed.

## Material and methods

### Animals, experimental design, and sampling

Fifty-four healthy Sprague Dawley rats obtained from the Laboratory Animal Housing Unit were males, 12 weeks old, and of initial weight of 220–250 g. Under a 12 h light/12 h dark cycle, rats have been kept in a well-ventilated room in a stainless steel case with food and water freely accessible. Two weeks before use in any trial, rats were adapted to laboratory conditions. Animals have been treated humanely, and ethical concerns are addressed in every respect. During the entire experiment, rats have been closely observed for distress, pain, injury, suffering, mucous membrane color, respiratory patterns, illness, and death.

Earlier in the experiment, the experimental animals were anesthetized by intramuscular injection of a cocktail of xylazine (5 mg/kg b.wt) and ketamine hydrochloride (50 mg/kg b.wt). Then, the hair of rats’ dorsal skin was clean-shaven until the epidermis fully appeared and then the skin was cleared using povidone iodine. Incised wound model was formed by scalpel on the dorsal skins following the protocol of Guan et al. [31]. An incision of 1-cm long was in the central dorsum skin layer using a scalpel. Each rat was individually placed in a cage after wounding and was given sterilized chow and water, in order to prevent bacterial contamination. Rats have not been given pain medication after the skin injury to avoid the influence of analgesics on wound healing which could add a significant variable in analyzing animal-derived data for relevance to humans [32–34]. But, we reduced pain stress on rats by single caging, using soft bedding, easily accessible food and water, and avoid rough handling. The rats were anesthetized and sacrificed via cervical dislocation, and 1*:*5 × 2 cm^2^ specimens were collected from the wounded areas at 30 min, 2 h, 6 h, 12 h, 1 day, 3 days, 5 days, and 7 days (6 rats in each group) post-wounding. The specimens were immediately stored at − 80 ºC until gene expression analysis. The blood samples were taken from the retro-orbital plexus. The serum was obtained via centrifugation for 10 min at 3000 rpm. The resultant serum samples were used for the evaluation of TNFα, IL1β, and NO levels. As a control, skin specimens and blood samples from six rats without incision were collected. The data of 0 h denote the results of the control rats.

### Quantitative analysis of HMGB1, TLR2, and TLR 4 relative gene expression

Total RNA has been isolated from the skin tissue with the RNA purification kits as directed by the company. In accordance with manufacturer guidelines, cDNA first-strand was reverse transcribed from 1 μg of the total RNA reverse transcription kit. These samples were frozen at 80ºC to be used in real-time PCR to determine gene expression levels. A Rotor-Gene Q cycler (Qiagen Hilden, Germany) and a QuantiTect® SYBR® Green PCR Kit (Qiagen Hilden, Germany) together with forward and reverse primers specific to each gene were used in quantitative real-time PCR. The 25-µl PCR mixture contains cDNA (2 μl), primer (2 μl), 2 × SYBR Green PCR Master Mix (12.5 μl), and RNase-free water (8.5 μl). Amplification conditions were 15 min of initial activation at 95ºC, 40 cycles at 94ºC for 15 s for denaturation, 30 s of annealing at 58ºC, and finally 30 s of elongation at 72ºC, respectively. The comparative 2^−ΔΔCt^ method was used to calculate relative fold changes [35]. Real-time PCR thermal profile and Primer sequences are shown in Table 1.[36, 37]

**Table 1:** Oligonucleotide primer sequences and real-time PCR conditions

Gene name		Primer sequences	Reaction conditions	Reference
HMGB1	F	5′- TGATTAATGAATGAGTTCGGGC-3′	94 °C-30 s/59 °C-30 s/ 72 °C-60 s (27 cycles)	^1^
	R	3′- TGCTCAGGAAACTTGACTGTTT-5′		
TLR2	F	5′- TCTGAGTTCCGTGACATAGG-3′	95◦C-15 s/59◦C-40 s/72◦C-40 s (40 cycles)	^2^
	R	3′- AGATGTAACGCAACAGATTC-5′		
TLR4	F	5′- GTGAGCATTGATGAGTTCAG-3′	95◦C-15 s/59◦C-40 s/72◦C-40 s (40 cycles)	^2^
	R	3′- CATCTAATGATTGATAAGGATT-5′		
GAPDH	F	5′- ATCAACGACCCCTTCATTGACC -3′	94◦C-40 s/59◦C-40 s/72◦C-40 s (35 cycles)	^2^
	R	3′- CCAGTAGACTCCACGACATACTA -5′		

*F* forward primer, *R* reverse primer, *HMGB1* high-mobility group box-1, *TLR2* toll-like receptors 2, *TLR4* toll-like receptors 4, *GAPDH* glyceraldehyde-3-phosphate dehydrogenase

### Evaluation of TNF-α, IL-6, and NO serum levels

Serum levels of TNF-α (Cat. no. STA00D) and IL-6 (Cat. no. S6050) were estimated.

using a rat enzyme-linked immunosorbent assay (ELISA) kit (Quantikine Co., Minneapolis, MN, USA) in line with the manufacturer’s instructions. The NO level was detected using ready kits (Abcam, Co., Cambridge, MA, USA; ab65328) consistent with the manufacturer’s protocols.

### Data analysis

The SPSS/PC + 2001 computer program was used for the statistical analysis of the existing study data. The statistical model used here was one-way ANOVA with LSD post hoc test. Data are shown as means ± the standard error. The minimum significance level was set as *p* < 0.05. Correlation between the estimated parameters was assessed by Pearson’s correlation coefficient (r). The curve estimation analysis between the relative expression of different genes and levels of serum proinflammatory cytokines and nitric oxide and the time after wounding was performed to develop the best mathematical model function. Four mathematical model functions including linear, quadratic, cubic, and exponential were investigated. The best mathematical models were considered curves with the highest determination coefficient (R^2^).

## Results

### HMGB1, TLR2, and TLR4 mRNA relative expression in skin tissues

**Fig. 1:**
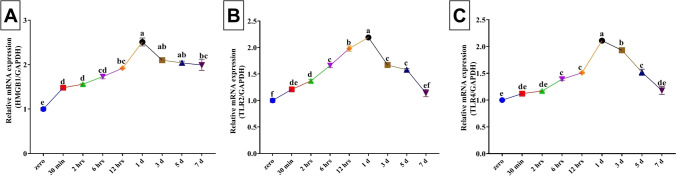
Changes in the relative expression of high-mobility group box-1 (HMGB1) and toll-like receptors 2 and 4 (TLR2 and 4) in the rat wound tissue collected at different time points. The expression abundance of genes mRNA was normalized against the internal control gene GAPDH using quantitative real-time PCR technique. Values are mean ± SE. *n* = 6. Post-wounding time points carrying different letters (a, b, c, d, e, and f) are significantly different at *P* < 0.05

As demonstrated in Fig. 1, after incision, there was an increase in HMGB1, TLR2, and TLR4 mRNA expressions in a time dependent manner till the 1^st^ day where the peak level was achieved for the three tested genes (2.51 ± 0.09, 2.19 ± 0.02, and 2.11 ± 0.02, respectively) compared with the zero time (1.00 ± 0.04, 1.00 ± 0.04, and 1.00 ± 0.12, respectively). For TLR2 and TLR4, a time-dependent decrease in their mRNA expression level was recorded from the 3^rd^ day to the 7^th^ day where a non-significant change was recorded at the 7^th^ day compared to the zero time. On the other hand, the HMGB1 upregulation persist until the 7^th^ day (1.99 ± 0.12) with a significant difference from the zero time.

### Changes in serum levels of TNF-α, IL-6, and NO

**Fig. 2:**
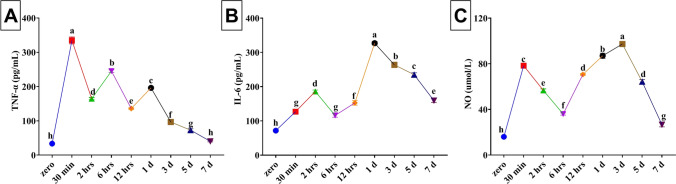
Changes in the serum levels of tumor necrosis factor α (TNF-α), interleukin 6 (IL-6), and nitric oxide (NO) collected at different time points from rats after wounding. Values are mean ± SE. Post-wounding time points carrying different letters (a, b, c, d, e, f, g, and h) are significantly different at *P* < 0.05

The serum TNF-α levels peaked within 30 min after injury (334.67 ± 10.14) compared to the zero time (33.67 ± 0.62) (Fig. 2A). Despite the significant (*P* < 0.001) decrease in the TNF-α level in the wounds 2 h–5 day old compared to the peak recorded at 30-min wound, but the statistical significance still exists compared to that in the control group (zero time). Nevertheless, at the 7^th^ day, no significant differences existed in the TNF-α serum level compared to the control group.

For IL-6, throughout the 7 days, significant (*P* < 0.001) increases were recorded with the peak achieved at the 1^st^ day post-wounding (327.00 ± 5.12) compared to the zero time (71.67 ± 3.09) (Fig. 2B). Moreover, at the 7^th^ day, the statistical significance (*P* < 0.001) was not lost with mean values of 159.33 ± 5.72.

As presented in Fig. 2C, there was a significant (*P* < 0.001) increase in the NO level peaked at the 3^rd^ day after wounding with mean values of 97.33 ± 1.25 compared to the zero time (16.00 ± 0.82). In spite of the significant (*P* < 0.001) decrease in the NO level in the wounds 5 and 7 day old compared to the peak recorded at the 3^rd^ day wound but the statistical significance still exist compared to that in the control group (zero time).

### Mathematical models for estimation of the age of the wound

A curve estimation analysis between the age of wound and the estimated biomarkers including relative expression of healing-related genes in the wound and serum cytokines in rats was performed. The mathematical models of the three genes (HMGB1, TLR2, and TLR4) and the serum biochemical indicators (TNFα, IL-6, and NO) comprising linear, quadratic, cubic, and exponential equations with their determination coefficients (R^2^) were proven by SPSS program. Mathematical models with the highest R^2^ values were considered optimal for estimating the wound age and are displayed in Table 2. The curves fit between the age of wound, and the estimated indicators are shown in Figs. 1 and 2. It was apparent that the analyzed genes in the wound tissues showed higher *R*^2^ values rather than the serum biochemical indicators. Among analyzed genes, TLR2 achieved the highest *R*^2^ value, while that of IL-6 was the highest among estimated serum indicators.

**Table 2:** Optimum mathematical models and equations used to describe the relationship between the time after wounding (X) and the estimated biomarkers including relative expression of healing-related genes in the wound and serum cytokines in rats (Y)

Biomarker	Model	Equation	R^2^
HMGB1	Cubic	*y* = 0.798 + 0.244*x* + 0.018 *x*^2^ − 0.003 *x*^3^	0.821
TLR2	Cubic	*y* = 0.832 + 0.086*x* + 0.065 *x*^2^ − 0.008 *x*^3^	0.872
TLR4	Cubic	*y* = 1.327 + 0.443*x* + 0.173 *x*^2^ − 0.014 *x*^3^	0.859
TNFα	Cubic	*y* = − 101.895 + 237.187*x* -49.037 *x*^2^ + 2.736 *x*^3^	0.562
IL-6	Cubic	*y* = 127.925–56.258*x* + 25.169 *x*^2^ − 2.052*x*^3^	0.647
NO	Cubic	*y* = 38.811–9.645*x* + 6.595 *x*^2^ − 0.623*x*^3^	0.506

*HMGB1* high-mobility group box-1, *TLR2* toll-like receptors 2, *TLR4* toll-like receptors 4, *TNFα* tumor necrosis factor alpha, *IL-6* interleukin-6, *NO* nitric oxide

### Correlation between the estimated parameters

As demonstrated in Table 3, a strong positive correlation was evident between the relative expression level of HMGB1 and that of TLR2 and TLR4 as well as IL-6 serum level. In addition, there was an intermediate positive correlation between HMGB1 in wound tissue and NO in the serum of rat after wounding. TLR2 is strongly correlated with TLR4 and intermediately correlated with serum levels of both IL-6 and NO. In addition, TLR4 is strongly correlated with IL-6 and NO serum levels. Of note, a strong correlation was detected between IL-6 and NO serum levels. Nonetheless, no significant correlation was estimated between TNF-α level and any of the estimated parameters except weak correlation with the NO level.

**Table 3:** Correlation coefficient (r) between expression of healing-related genes in the wound and serum cytokines in rats

	HMGB1	TLR2	TLR4	TNF	IL6	NO
HMGB1	1	0.769**	0.852**	− 0.004	0.847**	0.591**
TLR2		1	0.849**	0.215	0.673**	0.628**
TLR4			1	0.027	0.870**	0.760**
TNF-α				1	− 0.037	0.381*
IL6					1	0.732**
NO						1

*HMGB1* high-mobility group box-1, *TLR2* toll-like receptors 2, *TLR4* toll-like receptors 4, *TNFα* tumor necrosis factor alpha, *IL-6* interleukin-6, *NO* nitric oxide. *Correlation is significant at the 0.05 level.**Correlation is significant at the 0.01 level

## Discussion

The determination of wound age continues to be a challenge in forensic medicine [38]. Hence, several approaches and techniques were used to determine the vitality and duration of wounds. For instance, in various studies that investigated tools for determining wound’s age in human tissues taken from autopsy cases and their results are not used in forensic as evidence at court, traditional histological wound features are applied in addition to several immunohistochemical markers [5, 7, 39, 40]. In order to achieve this, excisions of the skin are made, in which the actual damage can be evaluated mostly at the dermo-subcutaneous junction [3, 41]. Nevertheless, it is not ethical to excise skin injuries in living individuals [1]. Apart from morphological methods, there were numerous attempts to establish biochemical methods for the evaluation of vitality markers in wound fluids or tissue extracts of wounds [42, 43]. Immunological tests such as ELISA are one of the biochemical methods that gave valuable results with the analysis of pro-inflammatory cytokines in wounds [4, 44]. Moreover, molecular biology could provide valuable tools to carry out studies on wound timing by providing definite information of the stage of the cell activation and which mediator is to be synthesized [45]. For instance, the use of RT-PCR has already given some interesting results for the correlation of cytokines levels and timing of different types of wounds [46, 47]. Furthermore, combining proteomic and genomic technologies in earlier studies provided important details about the interconnected processes that could define the age of wounds [48, 49]. Importantly, to establish the chronology of a wound using the aforementioned techniques, searching for more appropriate markers reliable for forensics is an extremely important issue [45].

In the current experiment, molecular techniques like RT-PCR were used to investigate the expression of target proteins, as it requires minute skin biopsies. In recent decades, transcripts were used as markers for the early phases of vitality [50, 51]. Because mRNA appears earlier than the protein that it encodes, mRNA evaluation using RT-PCR is more appropriate for estimating early wound age [52]. Moreover, the serum indicators could be a practical tool in living subjects for forensic practitioners. Thus, herein, we have followed the chronological changes in the levels of three analyzed genes in wound tissue and three biochemical indicators in serum up to 7 days post-wounding as the combined use of numerous indicators in the estimation of wounds age is considered a promising strategy [38].

In this study, the expression level of HMGB1, TLR2, and TLR4 was significantly increased in a time-dependent manner in the wound tissue up to the 7^th^ day where the statistical significance was lost for both TLR2 and TLR4 but persist for HMGB1. Moreover, our results displayed the strong correlation exists between the three tested analyzed genes and the high *R*^2^ value in the cubic mathematical regression model that was attained for all of them. Some cytokine and enzyme mRNAs have been investigated in recent years to determine the injury age [1, 3, 10, 20, 24]. But, there are no available reports on the chronological investigation of HMGB1, TLR2, and TLR4 gene expression post-wounding and how their levels correlated with each other in the wound environment.

Initially, HMGB1 is a multifunctional cytokine normally expressed in the nucleus of skin cells. But, it can be released into the extracellular space from damaged cells or from macrophages and monocytes in response to numerous stimuli like IL-1β and TNF-α in living individuals for skin regeneration and wound healing under physiological conditions [14]. Lower HMGB1 levels concomitant with delayed wound healing were detected in the diabetic patients and mice skin [53]. Thus, the significant upregulation of HMGB1 that recorded after 7 days of wound incision could be linked to the multiple sources of HMGB1 that associated with different stages of wound healing. HMGB-1 may passively escape from apoptotic, necrotic, and damaged cells [54] or be actively synthesized by immune cells including macrophages [55], natural killer cells [56], neutrophils, and mature dendritic cells [57], functioning as a cytokine-like molecule. HMGB1has roles inside the cytoplasm and as an extracellular damage-associated molecular pattern (DAMP) molecule [58]. HMGB1 can also interact with other TLR ligands and cytokines and triggers cells through several surface cell receptors, including TLR2 and TLR4, which work as an tissue damage sensor, causing inflammatory responses [59]. TLR2 and TLR4 are normally expressed in skin cells [60]. HMGB1 binding to TLR2/4 triggers a signaling cascade that leads to activation of NF-kB and then other proinflammatory cytokines, leukocyte adhesion molecules, and angiogenic factors in both hematopoietic and endothelial cells [61]. The positive association between the relative mRNA expression of HMGB1 and TLR2 and 4 has been recently confirmed in nasal brushing samples the study of Zhu et al. [62]. Of note, the Spearman’s correlation for HMGB1 relative expression in the wound tissue and IL-6 level in the serum was strong. In this regard, earlier evidence elucidated that HMGB1 on its own does not induce detectable IL-6 production. However, when the protein associates with one of several individual proinflammatory molecules (IL-1β, TLR4 ligand LPS), it potently enhances proinflammatory cytokine production [63, 64]. On the other hand, an intermediate positive correlation was found between HMGB1 in wound tissue and NO in the serum of rat after wounding. In this regard, Chakraborty et al. [27] suggested that the co-incubation of macrophages with rHMGB1 with either peptidoglycan or lipopolysaccharide significantly upregulated TLR2 and TLR4 surface receptors which in turn amplifies the NF-B activation and results in enhanced NO production.

Importantly, the peak of relative expression of HMGB1, TLR2, and 4 as well as serum level of IL-6 was delayed to the 1st day post-wounding. In this regard, the release of HMGB1 is postponed compared with TNF-α production and secretion in activated macrophages/ monocytes, with a 12–18-h lag phase [65]. In addition, Fang et al. [66] reported that thermal injury known to increase pro-inflammatory cytokine mRNA in different tissues also induced upregulated levels of HMGB1 mRNA 24 h after burning. However, the upregulation of HMGB1was significant till the 7^th^ day post-wounding implying the role of this protein in the different stages of wound healing. Similarly, Zhang et al. [67] reported that even 7 days after wounding, HMGB1 exists in the wound environment at a time in which the inflammatory phase of healing is well over. The same persistence of the significance increase was also recorded for both NO and IL-6 serum levels. This implies that these proteins determined in the inflammatory phase are implicated in inflammatory reactions and chemotaxis for neutrophil as well as immunosuppression, angiogenesis, and the chemotactic factor production for macrophages in the late proliferative stage [44, 67, 68].

In view of the fact that inflammation associated with injury can affect the entire system, herein, we supposed that systemic level of pro-inflammatory cytokine and NO production could be helpful in determining the age of wound together with the relative expression of other indicators in the wound microenvironment. Under physiological conditions in healthy subjects, TNF-α, IL-6, and NO are present at constant low serum levels because of their key roles in cellular homeostasis, immune surveillance, tissue regeneration, and the blood flow regulation [69–72]. But, their levels increases with tissue injury and activation of the blood cells in healthy subjected [73]. In the current study, serum TNF-, IL-6, and NO levels increased significantly with time after wounding, but the peak levels of each were reached at different times. In this regard, the peak level of TNF-α was obtained after 30 min of incision, but the peak levels of IL-6 and NO were obtained on the 1st and 3^rd^ days after incision, respectively. Comparably, the rapid release of TNF-α has been earlier reported in some studies that assessed the relative TNF-α expression in the wound microenvironment as an indicator to determine the time passed after wound [74, 75]. Thus, our study adds a new evidence of the possible use of TNF-α as an early phase wound age determinant, even serum level. Nevertheless, in the study of Sato and Ohshima [46], the peak mRNA levels for TNF-α were detected between 48 and 72 h in the wound microenvironment. While animal species intended to produce results as close to those obtained by humans are chosen for testing purposes, different findings may be derived from the use of various animal models [74]. On the other hand, the role of NO in the fibroblast collagen synthesis and contractile properties could elucidate such delay in the peak release at delayed stage [76].

The amount and the time of release of cytokines are affected by certain circumstances. These include malignant diseases, severe malnutrition, metabolic disorders, and medical treatment with glucocorticoid and cyto-static agents and chronic radiation exposure, which have a number of adverse effects on injury healing and delay of the cure process. In such instances, differences in cytokine values were shown [4]. Thus, further studies are highly recommended on cases associated with such conditions.

## Conclusion

From these results, we can conclude that HMGB1, TLR2, and 4 have comparable expression curves in the wound tissues possibly due to their correlated roles in the wound healing and their strong positive association. Mathematical models recognized for estimation of the wound age according to the expression level of each gene up to 7 days showed a high R2 value suggesting their use as a powerful determinant for the time after injury in forensic practices. Also, despite the significant increase in serum levels of TNF-α, NO, and IL-6, their use as wound age determinant could be less indicative than the relative expression of mRNA of targeted genes of wound tissue. Consequently, it is highly important to compare genomic results with serum levels of markers in order to find a serum marker that could help in forensic cases. Further studies about the expression of HMGB1 and TLR2 and 4 in traumatic human wounds are highly recommended to verify if the rat model can be useful also in human forensic cases. Furthermore, investigating the same determinants in short survival times during life and also in postmortem skin biopsies is highly needed.
